# Supplementary material for: Modeling the impact of national and regional lockdowns on the 2020 spring wave of COVID-19 in France
Source: Sci Rep. 2023 Feb 1;13:1834. doi: 10.1038/s41598-023-28687-w (PMC9890427; doi:10.1038/s41598-023-28687-w)
Supplement: Supplementary file 1 — Supplementary Information 1. [file 41598_2023_28687_MOESM1_ESM.pdf]

Modeling the impact of national and regional lockdowns on the 2020 spring wave of COVID-19 in France

Jonathan Roux[1], Clément Massonnaud[1,2], Vittoria Colizza[3], Simon Cauchemez[4], Pascal Crépey[1,*]

[1] RSMS - U 1309, ARENES - UMR 6051, EHESP, CNRS, Inserm, Université de Rennes, Rennes, France.

[2] Rouen University Hospital, Department of Biomedical Informatics, 76000 Rouen, France

[3] INSERM, Sorbonne Université, Institut Pierre Louis d'Epidémiologie et de Santé Publique IPLESP, F75012 Paris, France

[4] Mathematical Modelling of Infectious Diseases Unit, Institut Pasteur, UMR2000, CNRS, Paris, France

*pascal.crepey@ehesp.fr

Supplementary information

Supplementary Table S1 Impact of the change of the lockdown date on the number of hospital and ICU admissions, deaths, life years lost and quality-adjusted life years lost per 100,000 inhabitants compared to the observed lockdown date per French Region.

See Excel file

Supplementary Table S2 Impact of the change of the lockdown date on the regional occupation of ICU beds and the maximum ICU beds needed at the peak per French region

See Excel file

Supplementary Table S3 Maximum number of ICU beds available per region in March 2020

Region	Maximum number of ICU beds
**GES**	1219
**IDF**	2885
**BFC**	339
**ARA**	1150
**CVL**	270
**HDF**	827
**PAC**	980
**PDL**	456
**NOR**	492
**OCC**	967
**NAQ**	844
**BRE**	397
**France**	10 826

ICU : Intensive care unit. GES: Grand-Est, IDF: Ile-de-France, BFC: Bourgogne-Franche-Comté, ARA: Auvergne-Rhône-Alpes, CVL: Centre-Val de Loire, HDF: Hauts de France, PAC: Provence-Alpes-Côte d'Azur, PDL: Pays de la Loire, NOR: Normandie, OCC: Occitanie, NAQ: Nouvelle-Aquitaine, BRE: Bretagne.

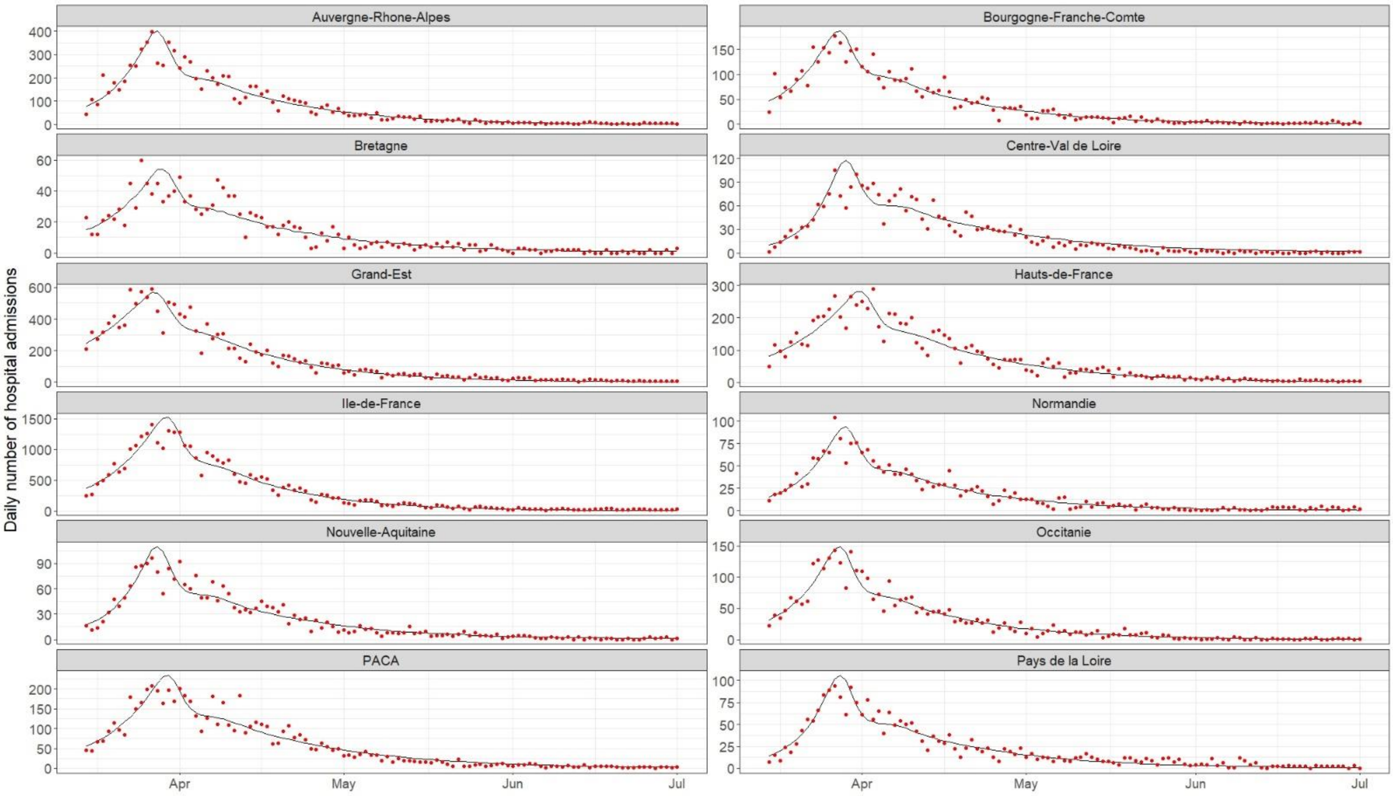

Supplementary Figure S1 Prediction of the number of new hospital admissions per French region between March 15 and July 1, 2020. The black line stands for the predicted values and the red dots for the observed values.

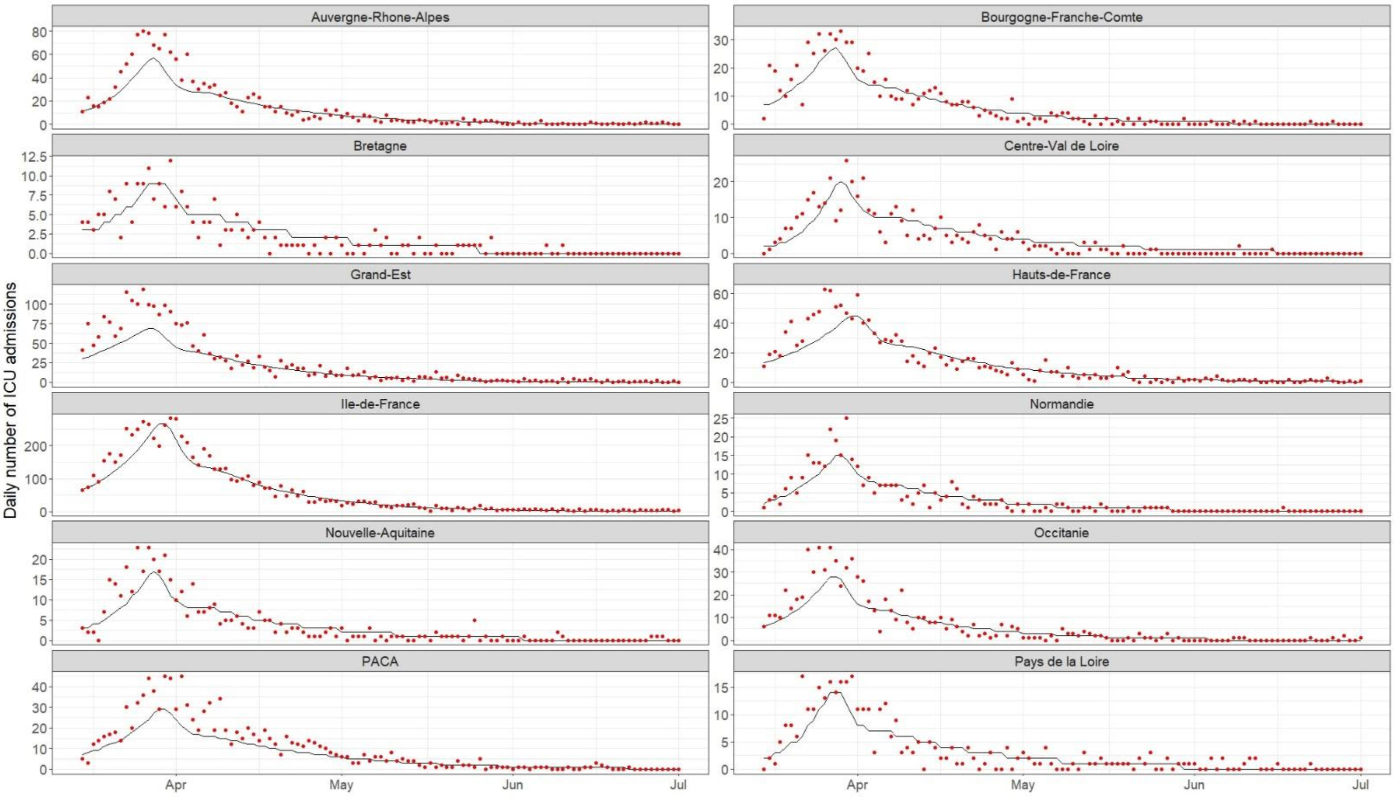

Supplementary Figure S2 Prediction of the number of new ICU admissions per French region between March 15 and July 1, 2020. The black line stands for the predicted values and the red dots for the observed values.

ICU: Intensive care unit.

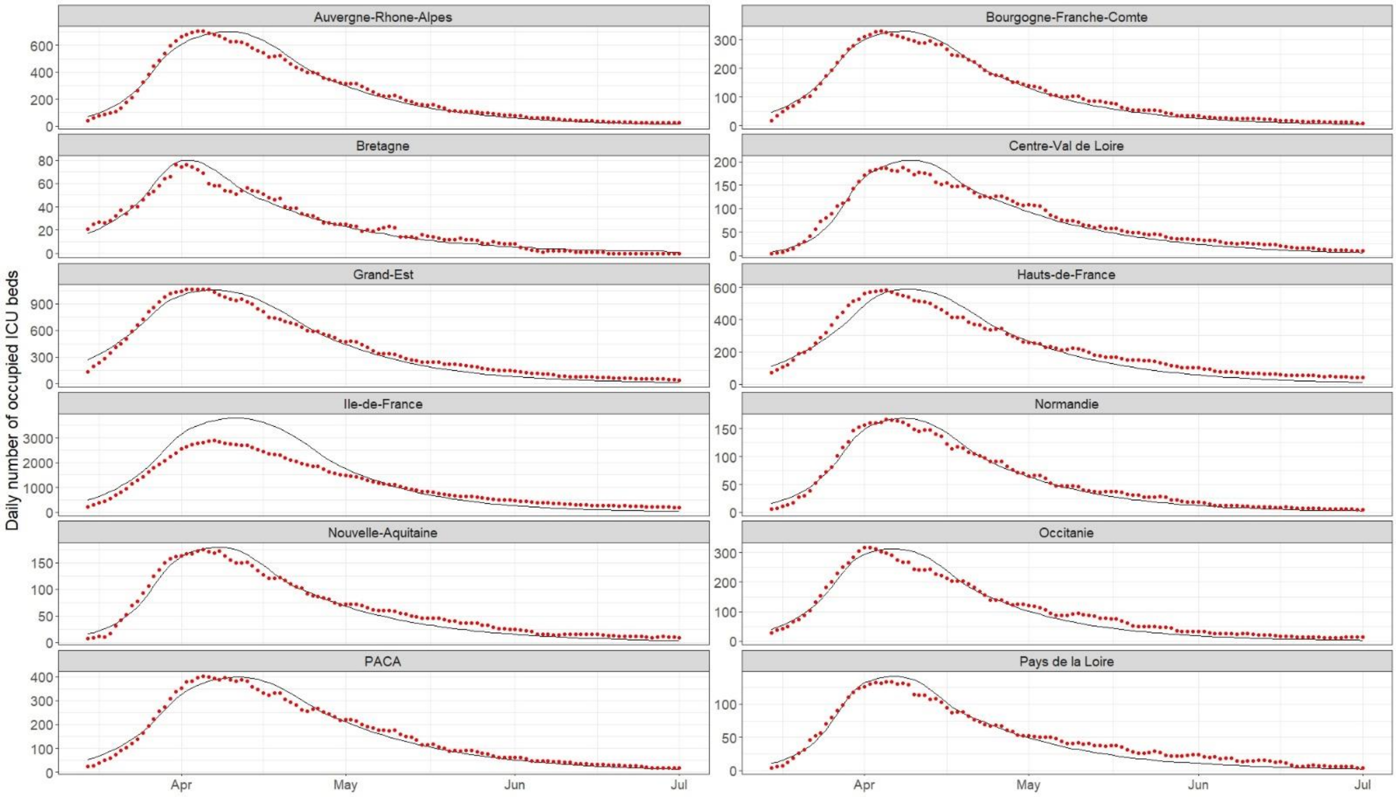

Supplementary Figure S3 Prediction of the number of occupied ICU beds per French region between March 15 and July 1, 2020. The black line stands for the predicted values and the red dots for the observed values.

ICU: Intensive care unit.

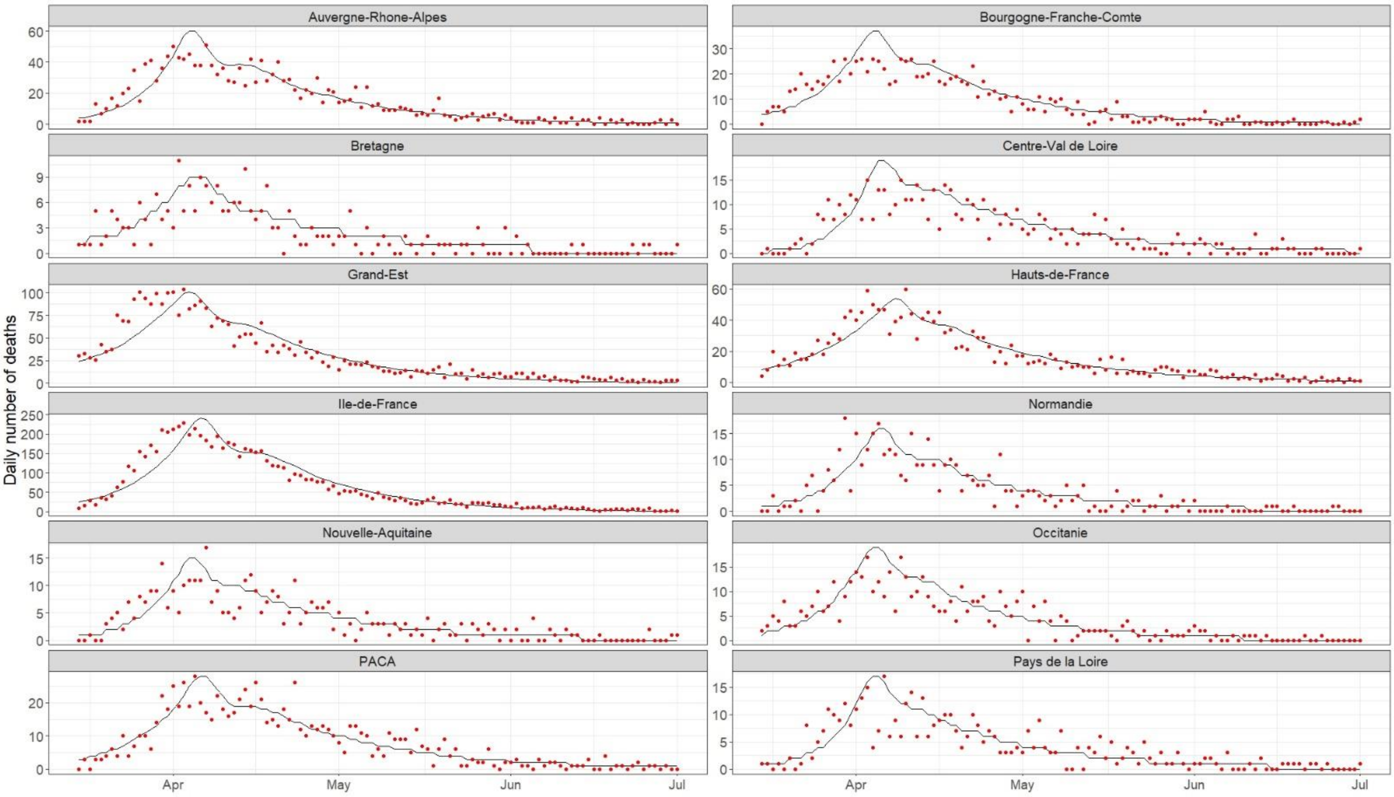

Supplementary Figure S4 Prediction of the number of new deaths per French region between March 15 and July 1, 2020. The black line stands for the predicted values and the red dots for the observed values.

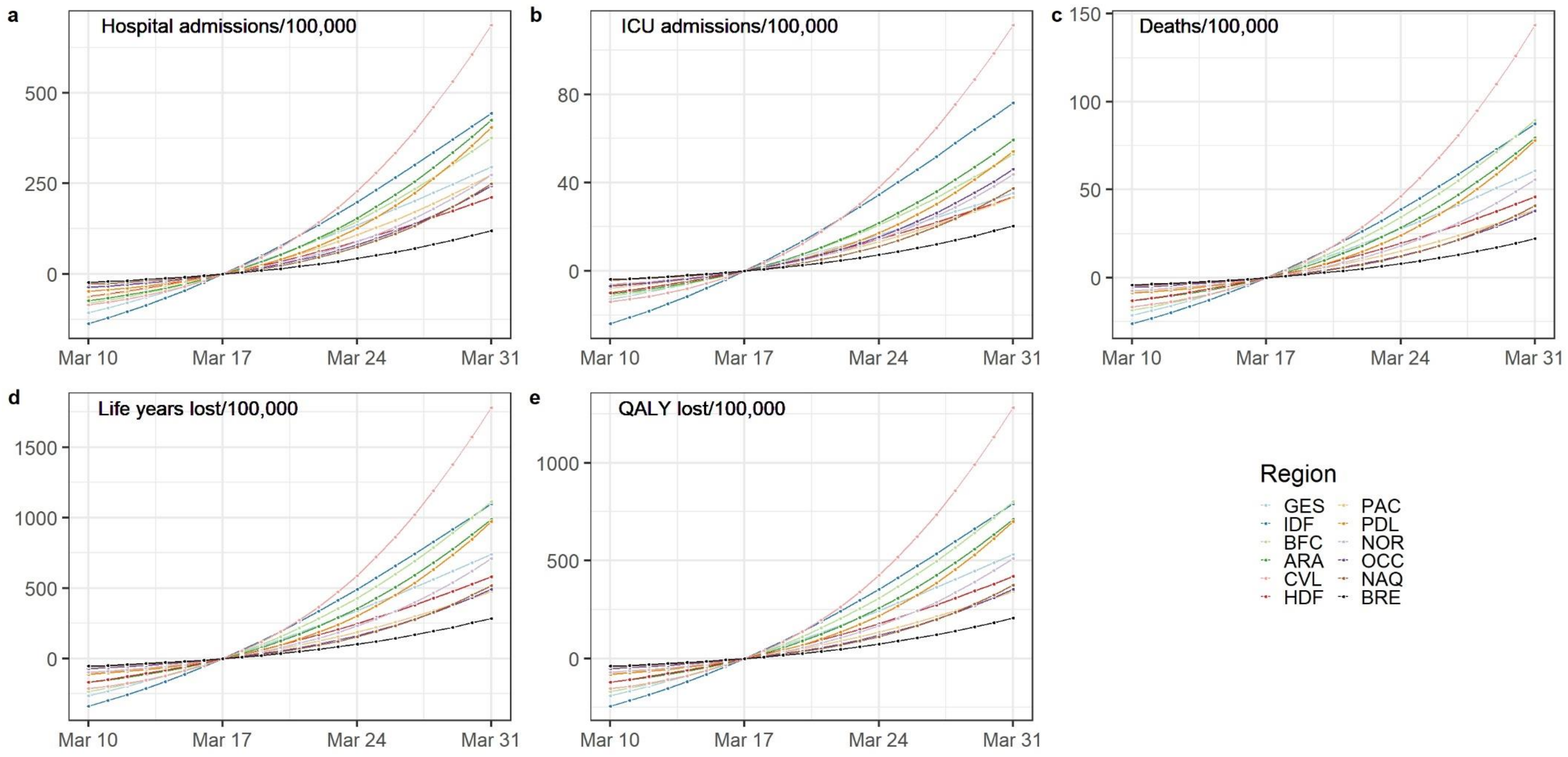

Supplementary Figure S5 Impact of the change of the lockdown date on the regional number of (a) hospital and (b) ICU admissions, (c) deaths, (d) life years and (e) quality-adjusted life years lost per 100,000 inhabitants compared to the observed lockdown date.

ICU: Intensive care unit. QALY : Quality-adjusted life years. GES: Grand-Est, IDF: Ile-de-France, BFC: Bourgogne-Franche-Comté, ARA: Auvergne-Rhône-Alpes, CVL: Centre-Val de Loire, HDF: Hauts de France, PAC: Provence-Alpes-Côte d'Azur, PDL: Pays de la Loire, NOR: Normandie, OCC: Occitanie, NAQ: Nouvelle-Aquitaine, BRE: Bretagne.

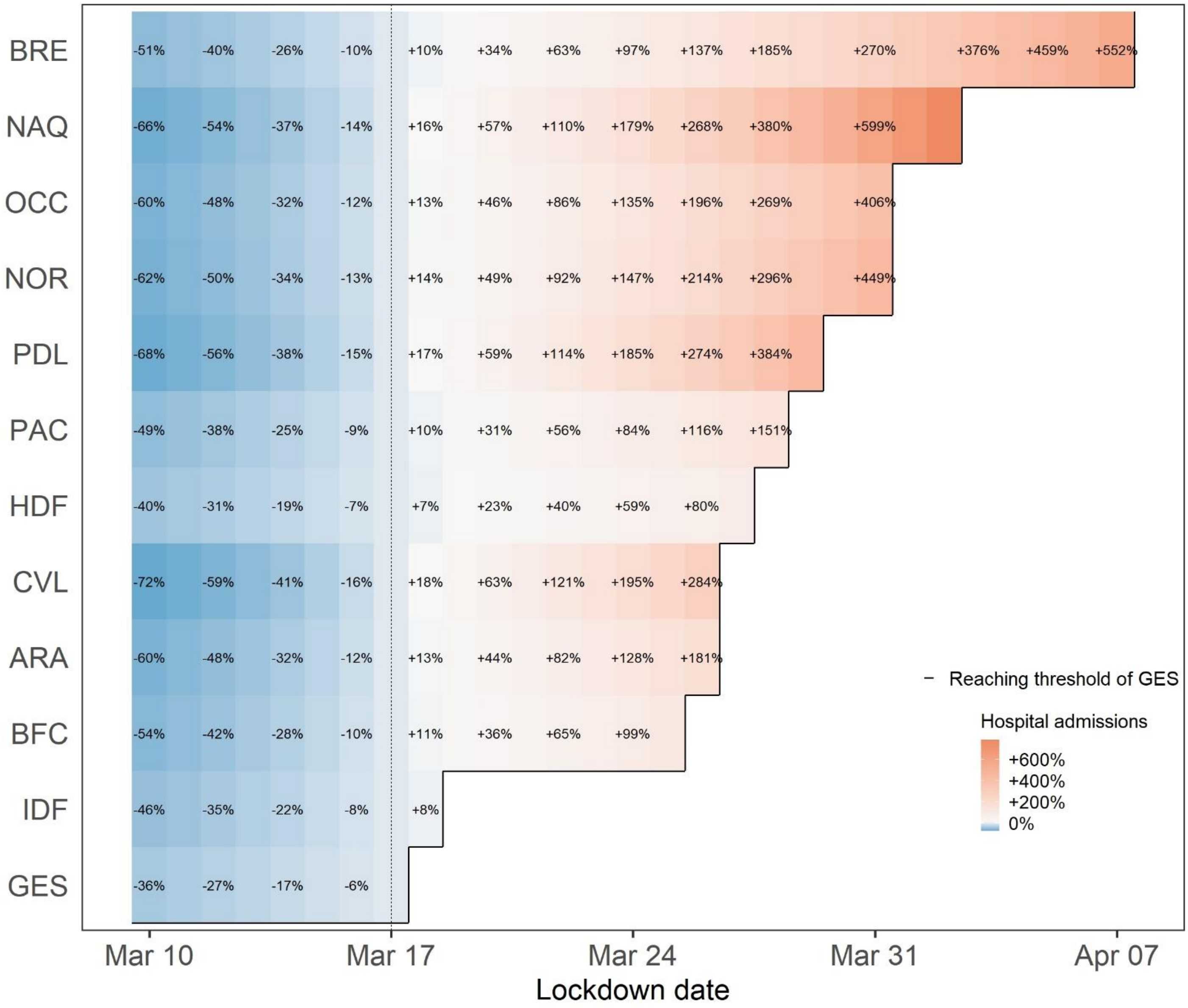

Supplementary Figure S6 Relative change in the number of hospital admissions (in %) according to lockdown date compared to the real lockdown for each French metropolitan region.

GES: Grand-Est, IDF: Ile-de-France, BFC: Bourgogne-Franche-Comté, ARA: Auvergne-Rhône-Alpes, CVL: Centre-Val de Loire, HDF: Hauts de France, PAC: Provence-Alpes-Côte d'Azur, PDL: Pays de la Loire, NOR: Normandie, OCC: Occitanie, NAQ: Nouvelle-Aquitaine, BRE: Bretagne.

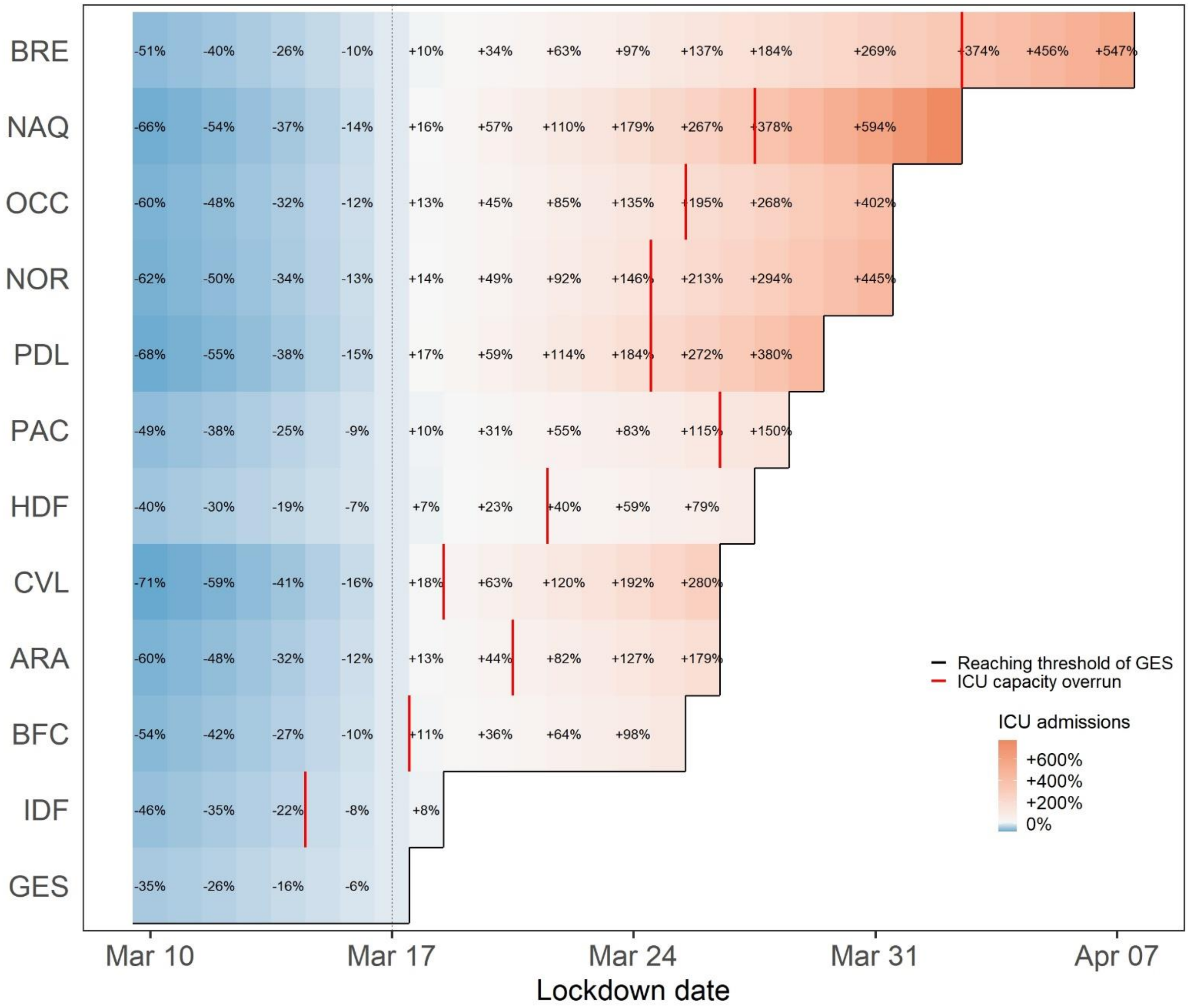

Supplementary Figure S7 Relative change in the number of ICU admissions (in %) according to lockdown date compared to the real lockdown for each French metropolitan region.

ICU: Intensive care unit. GES: Grand-Est, IDF: Ile-de-France, BFC: Bourgogne-Franche-Comté, ARA: Auvergne-Rhône-Alpes, CVL: Centre-Val de Loire, HDF: Hauts de France, PAC: Provence-Alpes-Côte d'Azur, PDL: Pays de la Loire, NOR: Normandie, OCC: Occitanie, NAQ: Nouvelle-Aquitaine, BRE: Bretagne.

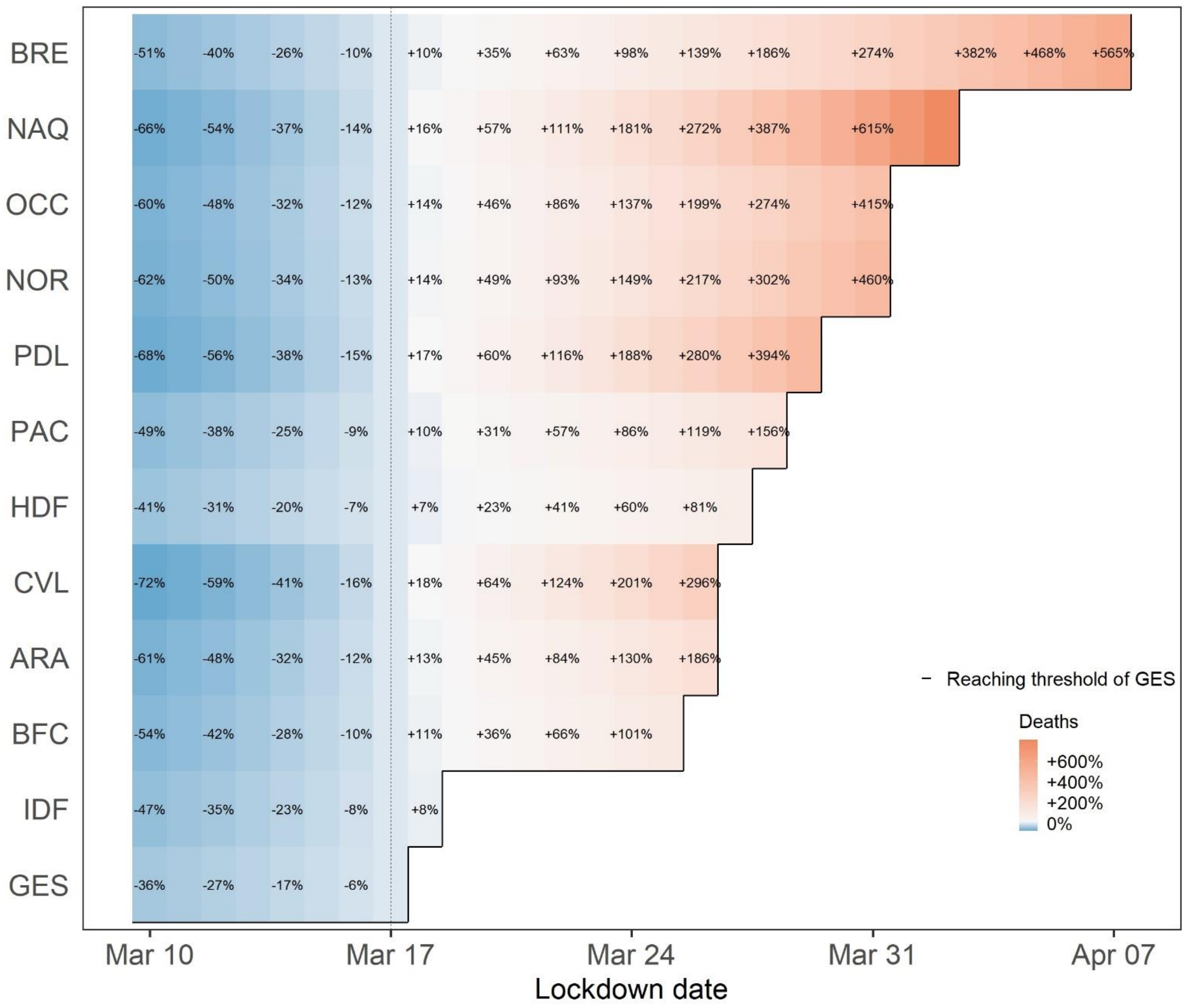

Supplementary Figure S8 Relative change in the number of deaths (in %) according to lockdown date compared to the real lockdown for each French metropolitan region.

GES: Grand-Est, IDF: Ile-de-France, BFC: Bourgogne-Franche-Comté, ARA: Auvergne-Rhône-Alpes, CVL: Centre-Val de Loire, HDF: Hauts de France, PAC: Provence-Alpes-Côte d'Azur, PDL: Pays de la Loire, NOR: Normandie, OCC: Occitanie, NAQ: Nouvelle-Aquitaine, BRE: Bretagne.

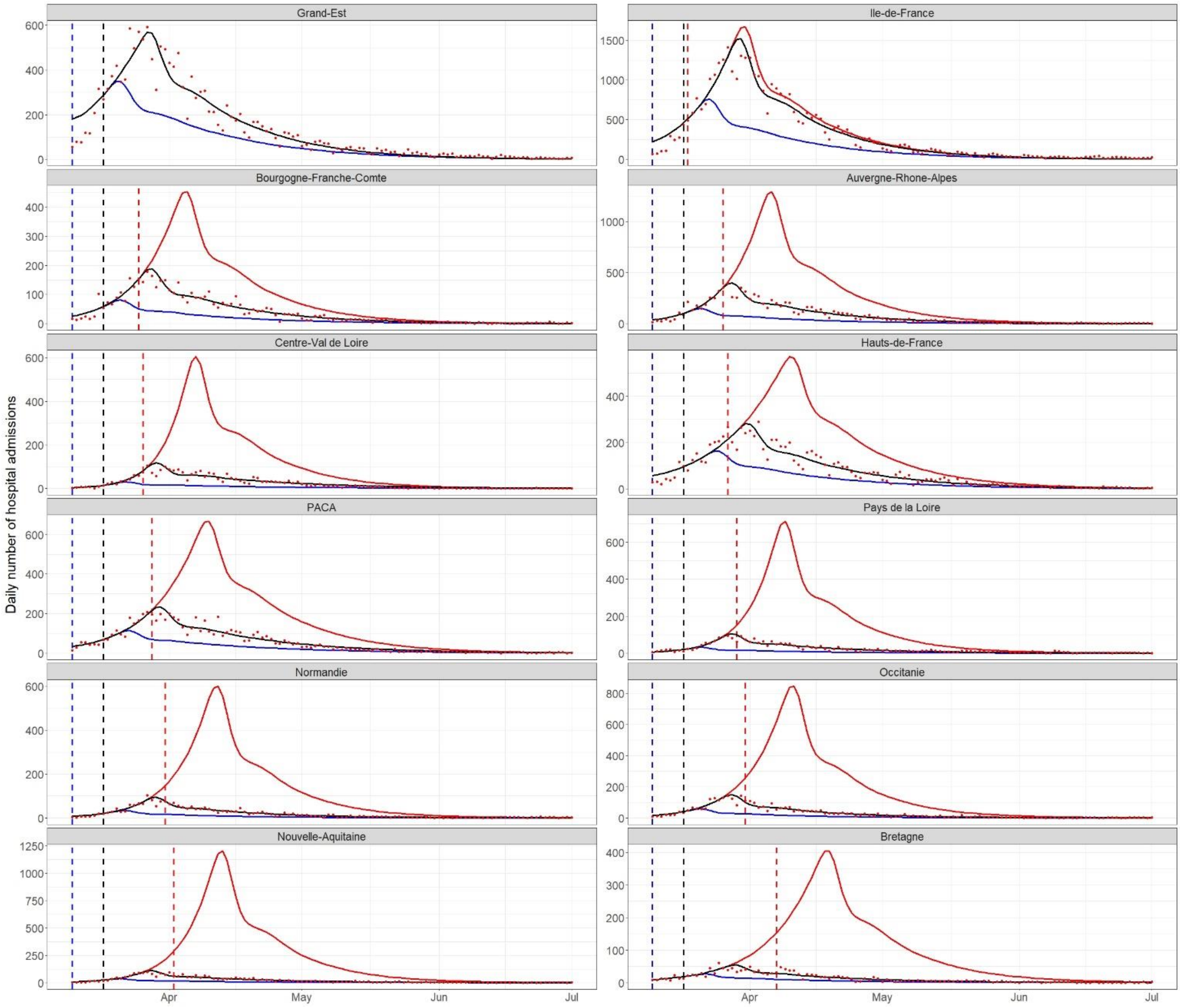

Supplementary Figure S9 Prediction of the number of new hospital admissions per French region between March 10 and July 1, 2020 with a lockdown start on March 10 (blue), March 17 (black) and GES threshold date (the date on which the estimated incidence of hospital admissions per 100,000 inhabitants in the region reached 6.50) (red). The dashed lines correspond to the starting dates of each lockdown and the red points to the observed values.

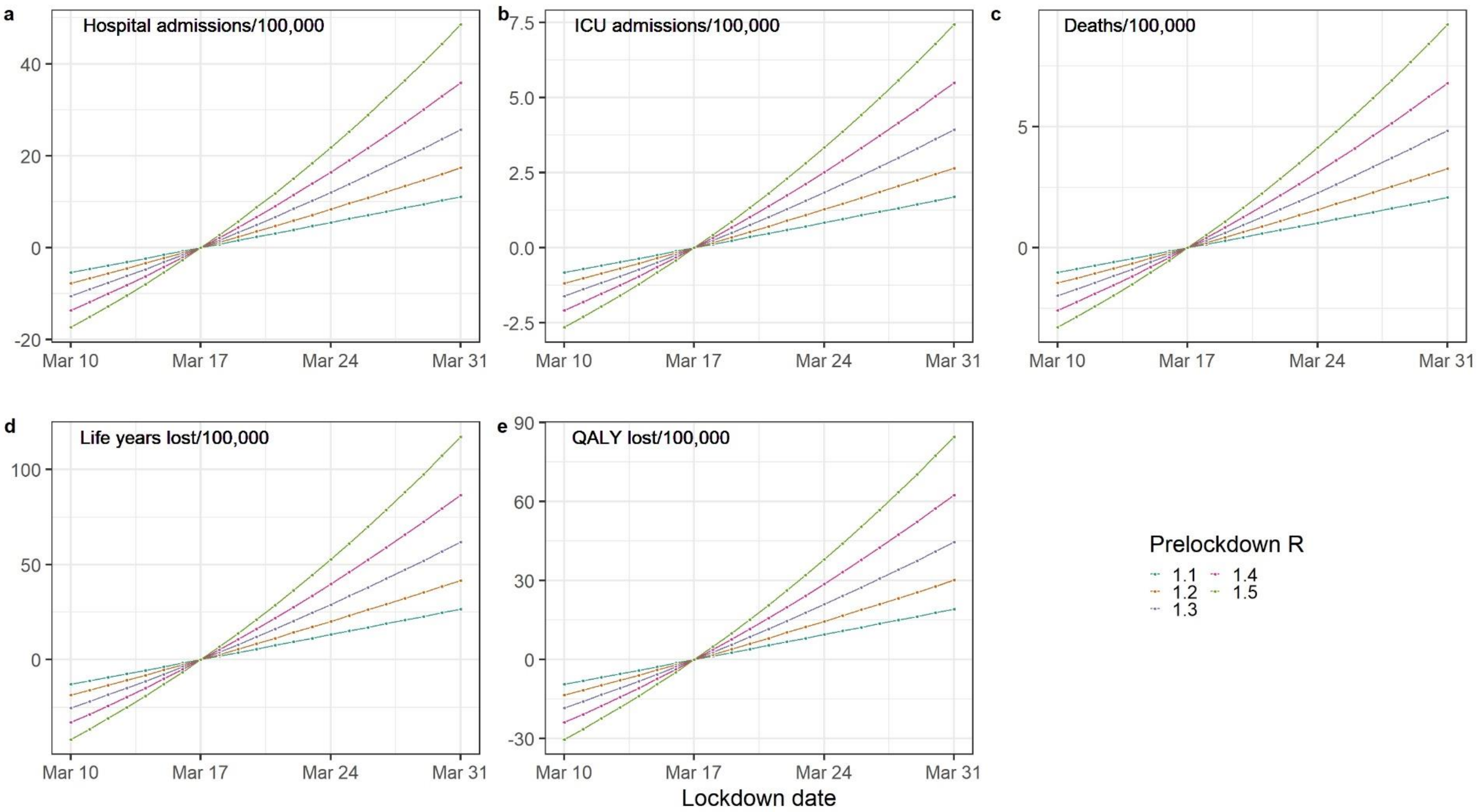

Supplementary Figure S10 Impact of the change of the lockdown date on the national number of (a) hospital and (b) ICU admissions, (c) deaths, (d) life years and (e) quality-adjusted life years lost per 100,000 inhabitants compared to the observed lockdown date according to the pre-lockdown value of the reproduction number $R_{prelockdown}$ ranging from 1.1 to 1.5.

ICU: Intensive care unit. QALY: Quality-adjusted life years.

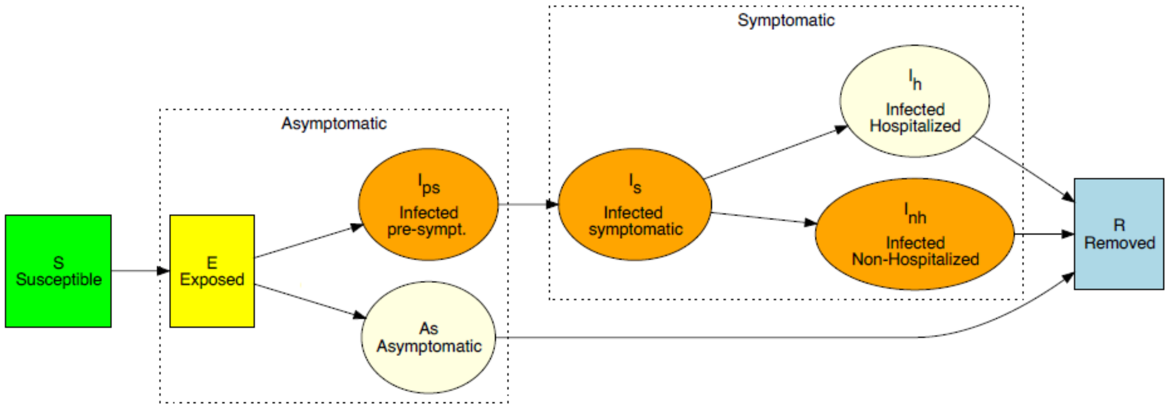

Supplementary Figure S11 Diagram of the SARS-CoV epidemiological model

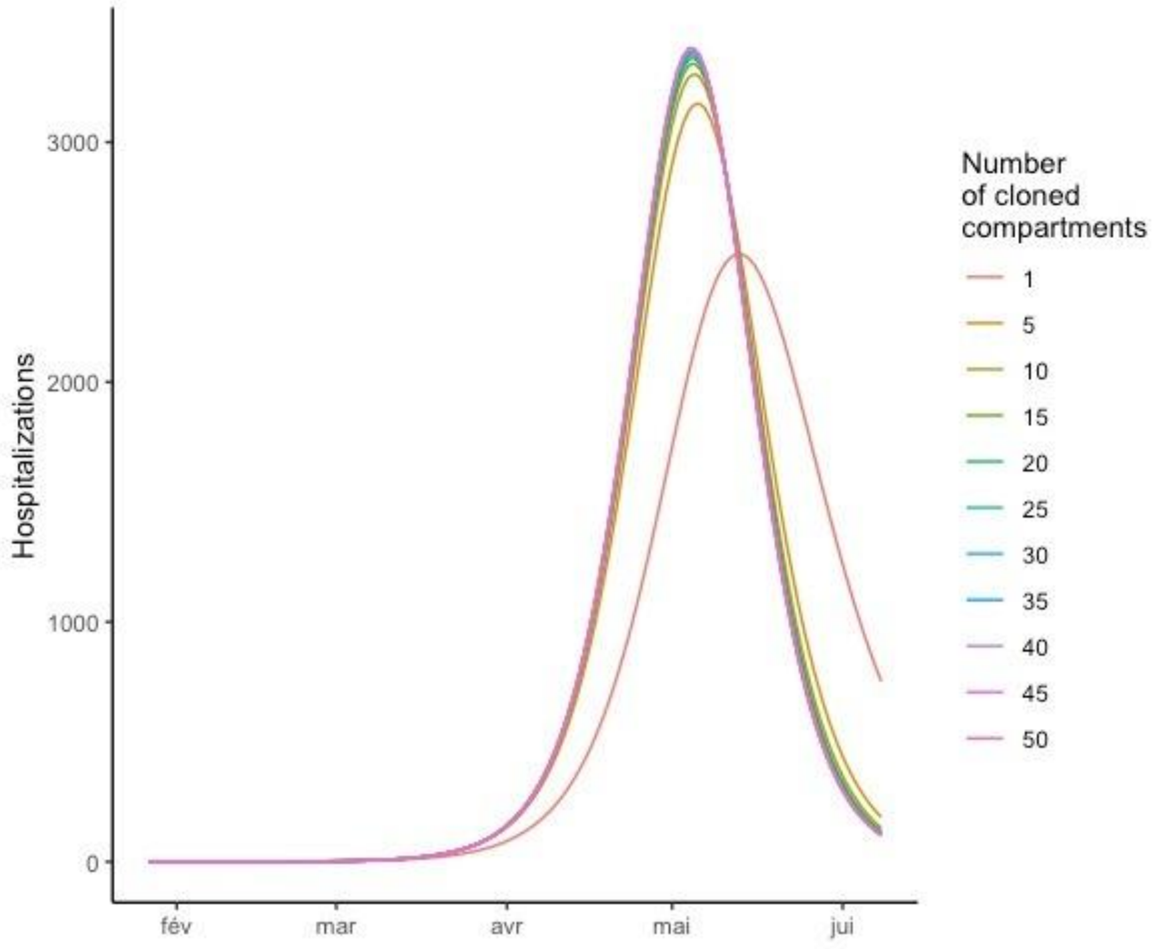

Supplementary Figure S12 Sensitivity analysis on the number of sub-compartments in each compartment of the transmission model

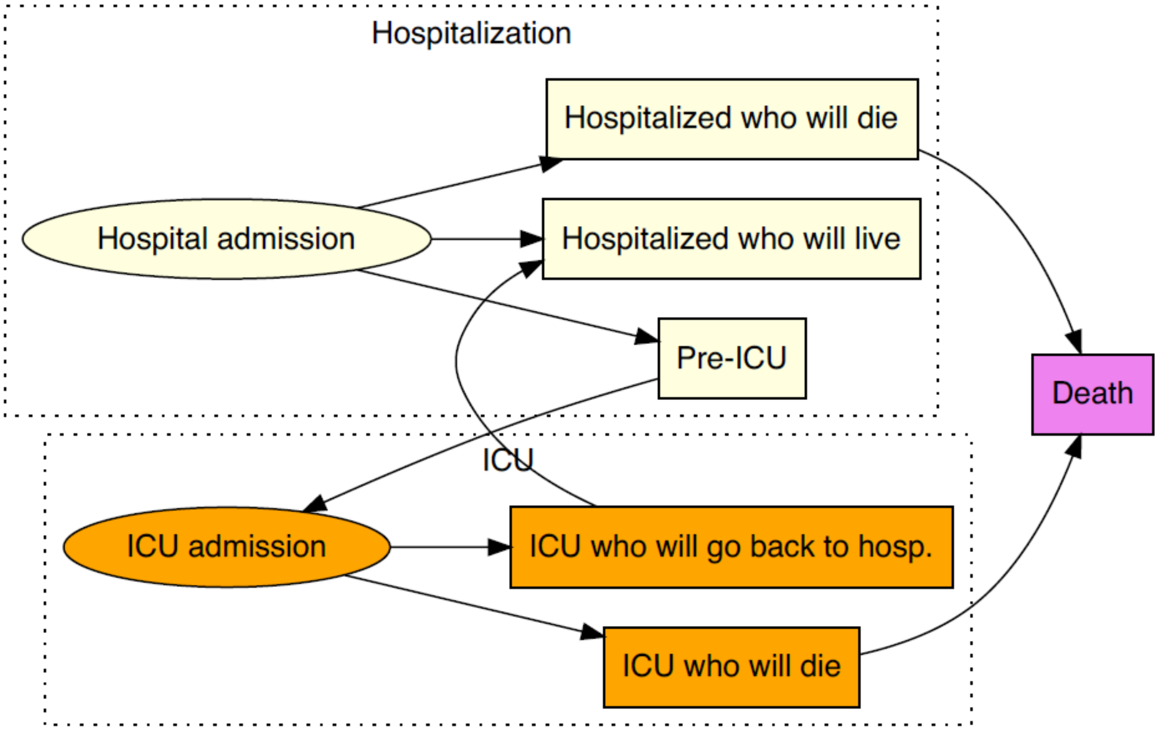

Supplementary Figure S13 Diagram of the care pathway in hospital settings of a hospitalized COVID-19 infected individual

ICU: Intensive care unit.

Equations of the model

The flows between the compartments are modeled with the following equations, with $f.A.B_i$ the flow from compartment A to compartment B, for age group i.

$$f.S.E_i = \beta.\left(\sum_j C_{ij}.\left(\frac{I_j}{N_j}\right)\right).S_i.sus_i$$

with i, j, the age categories, C the contact matrix, N the number of individuals, S the number of susceptible individuals, sus a susceptibility factor between 0 and 1, and I the number of infectious individuals, defined as:

$$I = \sum_{x \in X} I_x.\mathrm{inf_x}$$

with $X = \{I_{ps}, I_s, I_h, I_{nh}, A_s\}$, the set of infectious compartments, I_c the number of infected individuals in compartment c, and inf_c an infectivity factor between 0 and 1.

$$f.E.I_{ps_i} = E_i.progression.(1 - asymp_i)$$

$$f.E.A_{s_i} = E_i.progression.asymp_i$$

$$f.I_{ps}.I_{s_i} = I_{ps_i}.removal_{ps}$$

$$f.I_s.I_{h_i} = I_{s_i}.removal_s.hosp_i$$

$$f.I_s.I_{nh_i} = I_{s_i}.removal_s.(1 - hosp_i)$$

$$f.I_h.R_i = I_{h_i}.removal_h$$

$$f.I_{nh}.R_i = I_{nh_i}.removal_{nh}$$

$$f.A_s.R_i = A_{s_i}.removal_{as}$$

with *progression* the inverse of the average duration of pre-symptomatic incubation period, *removal$_{ps}$* the inverse of the average duration of pre-symptomatic infectious phase, *removal$_s$* the inverse of the average duration of pre-diagnostic phase, *removal$_h$* the inverse of the average duration of hospitalization, *removal$_{nh}$* the inverse of the average duration of diagnosed symptomatic phase, *removal$_{as}$* the inverse of the average duration of the asymptomatic phase.

Thus, we obtain the following set of ordinary differential equations:

$$\frac{dS_i}{dt} = -f.S.E_i$$

$$\frac{dE_i}{dt} = f.S.E_i - (f.E.I_{ps_i} + f.E.A_{s_i})$$

$$\frac{dI_{ps_i}}{dt} = f.E.I_{ps_i} - f.I_{ps}.I_{s_i}$$

$$\frac{dI_{s_i}}{dt} = f.I_{ps}.I_{s_i} - \left(f.I_s.I_{h_i} + f.I_s.I_{nh_i}\right)$$

$$\frac{dI_{h_i}}{dt} = f.I_s.I_{h_i} - f.I_h.R_i$$

$$\frac{dI_{nh_i}}{dt} = f.I_s.I_{nh_i} - f.I_{nh}.R_i$$

$$\frac{dA_{s_i}}{dt} = f.E.A_{s_i} - f.A_s.R_i$$

$$\frac{dI_{h_i}}{dt} = f.I_h.R_i + f.I_{nh}.R_i + f.A_s.R_i$$
